# Interpretation of Second Law of Thermodynamics in Extended Procedures for the Exploitation of the Entropy Inequality: Korteweg Fluids and Strain-Gradient Elasticity as Examples

**DOI:** 10.3390/e26040293

**Published:** 2024-03-27

**Authors:** Vito Antonio Cimmelli

**Affiliations:** Department of Mathematics, Computer Science and Economics, University of Basilicata, Via dell’Ateneo Lucano, 10, 85100 Potenza, Italy; vito.cimmelli@unibas.it

**Keywords:** entropy principle, dissipation inequality, weakly non-local constitutive equations, non-reversible direction axiom, extended Coleman–Noll procedure, strain-gradient elasticity

## Abstract

In continuum physics the dissipation principle, first proposed by Coleman and Noll in 1963, regards second law of thermodynamics as a unilateral differential constraint on the constitutive equations. In 1996, Muschik and Ehrentraut provided a rigorous proof of such an approach under the assumption that, at an arbitrary instant, $t_{0}$, in an arbitrary point, $P_{0}$, of a continuous system, the entropy production is zero if, and only if, $P_{0}$ is in thermodynamic equilibrium. In 2022, Cimmelli and Rogolino incorporated such an assumption in a more general formulation of the second law of thermodynamics. In this paper, we prove that the same conclusions hold if both the fundamental balance laws and their gradients are substituted into the entropy inequality. Such a methodology is applied to analyze the strain-gradient elasticity.

## 1. Introduction

### 1.1. Thermodynamic Compatibility of Korteweg Fluids

In this subsection, we prove that the classical Coleman–Noll method to obtain the consequences of the second law of thermodynamics on the mathematical models of continua fails in dealing with weakly non-local constitutive equations. To achieve this task, we consider the so-called Korteweg fluids, along with the density gradients in the stress tensor model capillarity effects. For such systems, in 1901, D. J. Korteweg postulated the constitutive equation [1],

(1) $$T_{ij}=\left(-p+\alpha \varrho_{,kk}+\beta \varrho_{,k}\varrho_{,k}\right)\delta_{ij}+\delta \varrho_{,i}\varrho_{,j}+\gamma \varrho_{,ij},$$

with $T_{ij}$ as the components of the stress tensor, $\varrho \left(x,t\right)$ as the mass density, and *p* as the pressure. The material functions α, β, γ, and δ depend on mass density and temperature, ϑ, and the symbol $f_{,i}$ in Equation (1) denotes the partial derivative of function *f* with respect to the spatial coordinate $x_{i}$.

Our aim here is not to develop a general thermodynamic model of such fluids, since an extensive study of them has been carried out in [2]. Here, we aim to illustrate the mathematical procedure used in [2], whose mathematical foundations will be analyzed more extensively in Section 2 and Section 3. Hence, in order to make this illustration more immediate, we make some simplifying assumptions that allow the reader to avoid cumbersome calculations. Readers who are interested in the complete analysis, without any approximation, are referred to [2].

If the heat supply is zero, the classical local balances of mass and energy,

(2) $$\varrho_{,t}+\varrho_{,i}v_{i}+\varrho v_{i,j}\delta_{ij}=0,$$

(3) $$\varrho \varepsilon_{,t}+\varrho \varepsilon_{,i}v_{i}-T_{ij}v_{i,j}+q_{i,i}=0,$$

hold, with $v_{i}$ and $q_{i}$ as the components of the velocity and of the heat flux, respectively, and ε as the specific internal energy.

Furthermore, the dissipation inequality,

(4) $$\varrho s_{,t}+\varrho s_{,i}v_{i}+J_{i,i}\geq 0,$$

with *s* as the specific entropy and $J_{i}$ as the components of the entropy flux, must be satisfied for an arbitrary thermodynamic process.

**Remark** **1.** *Here we do not discuss the problem of the definition of the entropy for general non-equilibrium processes but, according to the fundamental literature of early continuum thermodynamics *[3,4,5,6]*, we assume that the entropy production for an arbitrary material particle, c, of a continuum system in the absence of heat source can be expressed as*

(5) $$\frac{d}{dt}\int_{c}\rho sdc+\int_{\partial c}J_{i}n_{i}d\sigma =\frac{d}{dt}\int_{c}\rho sdc+\int_{c}J_{i,i}dc\;,$$

*where s is the internal entropy per unit of mass and $J_{i}$ is the local entropy flux through the boundary of c. In *[4,5]*, it is assumed that $J_{i}=q_{i}/\vartheta$, with ϑ the absolute temperature. Here, we do not specify the form of $J_{i}$ in order to incorporate both the classical expression, $q_{i}/\vartheta$, and also that postulated by Müller, $J_{i}=q_{i}/\vartheta +k_{i}$, wherein $k_{i}$ is known as entropy extraflux *[7]*. The arbitrariness of domain c and the regularity of the integrand function in (5), together with the requirement that the right-hand side of (5) is non-negative, lead to the inequality (4). For a recent discussion on non-equilibrium entropy, we refer the reader to *[8]* (see Section 4 therein).*

In order to prove that for such a class of materials the classical Coleman–Noll procedure [4] fails in investigating the thermodynamic compatibility, we apply it to the materials described by the set of constitutive equations,

(6) $$F=F\left(\varrho ,\varrho_{,k},\varepsilon ,\varepsilon_{,k}\right),$$

where *F* is an element of the set $\left\{T_{ij},q_{i},s,J_{i}\right\}$. The Korteweg fluids described by Equation (1) fall into this class if α=γ=0.

**Remark** **2.** *It is important to remark that this is a very simple model of Korteweg fluid. Moreover, we are aware that Korteweg fluids are only a subclass of more general higher-grade fluids, whose thermodynamic compatibility would require the introduction of a hyperstress *[9]*. Here, we follow the approach of Dunn and Serrin *[10]*, with the sole difference being that we do not introduce any energy extra flux accounting for the so-called interstitial working, as those authors do. Indeed, the focus of the present paper is the mathematical structure of weakly non-local constitutive theories, which will be developped in Section 2 and Section 3 below. The simple model analyzed here allow us to show to the reader that the classical exploitation procedures of the second law do not allow one to prove that they are compatible with thermodynamics without modifying the classical balances of energy or entropy.*

In the following section, we develop our calculations by using the index notation and not the compact one, which uses vectors. As we will see immediately, although a little bit longer, such a symbology allows one to better understand the mathematical structure of the quantities entering our equations and, in the case of tensors, the part of them that must vanish.

Due to (6), the local balance of energy (3) may be rewritten as

(7) $$\varrho \varepsilon_{,t}+\varrho \varepsilon_{,i}v_{i}-T_{ij}v_{i,j}+\frac{\partial q_{i}}{\partial \varrho }\varrho_{,i}+\frac{\partial q_{i}}{\partial \varrho_{,j}}\varrho_{,ji}+\frac{\partial q_{i}}{\partial \varepsilon }\varepsilon_{,i}+\frac{\partial q_{i}}{\partial \varepsilon_{,j}}\varepsilon_{,ji}=0.$$

The constitutive Equation (6) must be assigned in such a form to satisfy the inequality (4), namely,

(8) $$\begin{array}{c} \;\varrho \left(\frac{\partial s}{\partial \varrho }\varrho_{,t}+\frac{\partial s}{\partial \varrho_{,i}}\varrho_{,it}+\frac{\partial s}{\partial \varepsilon }\varepsilon_{,t}+\frac{\partial s}{\partial \varepsilon_{,i}}\varepsilon_{,it}\right)\\ \;+\varrho v_{i}\left(\frac{\partial s}{\partial \varrho }\varrho_{,i}+\frac{\partial s}{\partial \varrho_{,j}}\varrho_{,ji}+\frac{\partial s}{\partial \varepsilon }\varepsilon_{,i}+\frac{\partial s}{\partial \varepsilon_{,j}}\varepsilon_{,ji}\right)\\ \;+\frac{\partial J_{i}}{\partial \varrho }\varrho_{,i}+\frac{\partial J_{i}}{\partial \varrho_{,j}}\varrho_{,ji}+\frac{\partial J_{i}}{\partial \varepsilon }\varepsilon_{,i}+\frac{\partial J_{i}}{\partial \varepsilon_{,j}}\varepsilon_{,ji}\geq 0. \end{array}$$

Since both ϱ and ε enter the state space, their balances must be substituted into the inequality (8) in order to characterize the process that is being constrained.

**Remark** **3.** *Let us observe that, in order to prove Equation (3), the balance of linear momentum must be used. Thus, Equation (3) also includes information on such a balance.*

Then, the coupling of Equations (2), (3), and (8) yields

(9) $$\begin{array}{c} \;\varrho \frac{\partial s}{\partial \varrho_{,i}}\varrho_{,it}+\varrho \frac{\partial s}{\partial \varepsilon_{,i}}\varepsilon_{,it}+\langle \frac{\partial J_{i}}{\partial \varrho_{,j}}-\frac{\partial s}{\partial \varepsilon }\frac{\partial q_{i}}{\partial \varrho_{,j}}+\varrho v_{i}\frac{\partial s}{\partial \varrho_{,j}}\rangle \varrho_{,ij}\\ \;+\langle \frac{\partial J_{i}}{\partial \varepsilon_{,j}}-\frac{\partial s}{\partial \varepsilon }\frac{\partial q_{i}}{\partial \varepsilon_{,j}}+\varrho v_{i}\frac{\partial s}{\partial \varepsilon_{,j}}\rangle \varepsilon_{,ji}\;+\left(\frac{\partial s}{\partial \varepsilon }T_{ij}-\varrho^{2}\frac{\partial s}{\partial \varrho }\delta_{ij}\right)v_{i,j}\\ \;-\frac{\partial s}{\partial \varepsilon }\left(\frac{\partial q_{i}}{\partial \varrho }\varrho_{,i}+\frac{\partial q_{i}}{\partial \varepsilon }\varepsilon_{,i}\right)+\frac{\partial J_{i}}{\partial \varrho }\varrho_{,i}+\frac{\partial J_{i}}{\partial \varepsilon }\varepsilon_{,i}\geq 0, \end{array}$$

wherein the symbol $\langle F_{ij}\rangle$ denotes the symmetric part of the tensor function, $F_{ij}$.

The obtained inequality is linear with respect to the higher derivatives of the state functions $\varrho_{,it}$, $\varepsilon_{,it}$, $\varrho_{,ji}$, $\varepsilon_{,ji}$, and $v_{i,j}$, which are completely arbitrary [4].

**Remark** **4.** *The 27-dimensional vector $y=\left\{\varrho_{,it},\;\varepsilon_{,it},\;\varrho_{,ji},\;\varepsilon_{,ji},\;v_{i,j}\right\}$ is called by Muschik and Ehrentraut the precess-direction vector. With such a definition, these authors postulate the following amendment to the second law of thermodynamics: “Except in equilibria, reversible process-directions in the state space do not exist” *[11]*.*

Thus, the inequality (9) can be satisfied along arbitrary thermodynamic processes if, and only if, all the coefficients of the elements of y vanish, and the remaining part of (9) is non-negative. Then we have the following:

**Theorem** **1.** *The entropy inequality (8) is satisfied, whatever the thermodynamic process is, if, and only if, the following thermodynamic restrictions hold:*

(10) $$s=s\left(\varrho ,\varepsilon \right),$$

(11) $$\langle \frac{\partial J_{i}}{\partial \varrho_{,j}}-\frac{\partial s}{\partial \varepsilon }\frac{\partial q_{i}}{\partial \varrho_{,j}}\rangle =0,$$

(12) $$\langle \frac{\partial J_{i}}{\partial \varepsilon_{,j}}-\frac{\partial s}{\partial \varepsilon }\frac{\partial q_{i}}{\partial \varepsilon_{,j}}\rangle =0,$$

(13) $$T_{ij}=\varrho^{2}{\left(\frac{\partial s}{\partial \varepsilon }\right)}^{-1}\frac{\partial s}{\partial \varrho }\delta_{ij},$$

(14) $$\begin{array}{c} \;-\frac{\partial s}{\partial \varepsilon }\left(\frac{\partial q_{i}}{\partial \varrho }\varrho_{,i}+\frac{\partial q_{i}}{\partial \varepsilon }\varepsilon_{,i}\right)-\;\varrho^{2}\frac{\partial s}{\partial \varrho }v_{i,i}+\frac{\partial J_{i}}{\partial \varrho }\varrho_{,i}+\frac{\partial J_{i}}{\partial \varepsilon }\varepsilon_{,i}\geq 0. \end{array}$$

It is evident from Equations (10) and (13) that the stress tensor is completely local, and this leads to the conclusion that the constitutive Equation (1) is incompatible with the second law of thermodynamics. Several different proposals can be found in the literature to circumvent such a problem. All of them modify the energy balance (3) by introducing generalized energy or entropy fluxes [10,12]. An alternative approach, which changes the mathematical method but leaves the remaining balance equations unaltered, also regards the gradients of such equations as constraints, up to the order of the gradients entering the state space. That way, the number of constraints coincides with the number of independent thermodynamic variables. In this way, the first-order weakly non-local materials described by Equation (6) are compatible with the second law of thermodynamics [2]. In order to avoid lengthy and cumbersome calculations, here we prove such a statement under a semi-linear approximation, which consists of neglecting the terms constituted by a function defined on the state space multiplied by the product of two or more spatial derivatives. Thus, we calculate the gradients of Equations (2) and (3) by neglecting such terms. Thus, we obtain

(15) $$\varrho_{,it}+\varrho_{,ik}v_{k}+\varrho_{,k}v_{k,i}+\varrho_{,i}v_{k,j}\delta_{kj}+\varrho v_{k,ij}\delta_{kj}=0,$$

(16) $$\varrho \varepsilon_{,it}+\varrho \varepsilon_{,ki}v_{k}-T_{kj}v_{k,ji}+\frac{\partial q_{k}}{\partial \varrho }\varrho_{,ki}+\frac{\partial q_{k}}{\partial \varrho_{,j}}\varrho_{,jki}+\frac{\partial q_{k}}{\partial \varepsilon }\varepsilon_{,ki}+\frac{\partial q_{k}}{\partial \varepsilon_{,j}}\varepsilon_{,jki}=0,$$

wherein, according to the approximation established above, the partial derivatives of the Cauchy stress do not enter Equation (16).

The substitution of Equations (15) and (16) into (9) leads to the extended inequality

(17) $$\begin{array}{c} \;\langle \frac{\partial s}{\partial \varepsilon_{,i}}\frac{\partial q_{k}}{\partial \varrho_{,j}}\rangle \varrho_{,jki}+\langle \frac{\partial s}{\partial \varepsilon_{,i}}\frac{\partial q_{k}}{\partial \varepsilon_{,j}}\rangle \varepsilon_{,jki}\;+{\langle \frac{\partial s}{\partial \varepsilon_{,i}}T_{,kj}+\varrho^{2}\frac{\partial s}{\partial \varrho_{,i}}\delta_{,kj}\rangle }_{ij}v_{k,ij}\\ \;+\langle \frac{\partial J_{i}}{\partial \varrho_{,j}}-\frac{\partial s}{\partial \varepsilon }\frac{\partial q_{i}}{\partial \varrho_{,j}}+\varrho v_{i}\frac{\partial s}{\partial \varrho_{,j}}\;-\varrho v_{j}\frac{\partial s}{\partial \varrho_{,i}}\rangle \varrho_{,ij}\;+\langle \frac{\partial J_{i}}{\partial \varepsilon_{,j}}-\frac{\partial s}{\partial \varepsilon }\frac{\partial q_{i}}{\partial \varepsilon_{,j}}+\varrho v_{i}\frac{\partial s}{\partial \varepsilon_{,j}}-\varrho v_{j}\frac{\partial s}{\partial \varepsilon_{,i}}\rangle \varepsilon_{,ji}\\ \;+\left(\frac{\partial s}{\partial \varepsilon }T_{ij}-\varrho^{2}\frac{\partial s}{\partial \varrho }\delta_{ij}-\frac{\partial s}{\partial \varrho_{,j}}\varrho_{,i}-\frac{\partial s}{\partial \varrho_{,k}}\varrho_{,k}\delta_{ij}\right)v_{i,j}\\ \;-\frac{\partial s}{\partial \varepsilon }\left(\frac{\partial q_{i}}{\partial \varrho }\varrho_{,i}+\frac{\partial q_{i}}{\partial \varepsilon }\varepsilon_{,i}\right)+\frac{\partial J_{i}}{\partial \varrho }\varrho_{,i}+\frac{\partial J_{i}}{\partial \varepsilon }\varepsilon_{,i}\geq 0, \end{array}$$

wherein the symbol ${\langle F_{kij}\rangle }_{ij}$ denotes the symmetric part of the tensor function, $F_{kij}$, with respect to the couple, (i,j). In such a case, the entropy inequality is linear in the higher derivatives $\varrho_{,ij}$, $\varepsilon_{,ij}$, and $v_{i,j}$, and in the highest derivatives, i.e., the spatial derivatives whose order is the highest one, $\varrho_{,ijk}$, $\varepsilon_{,ijk}$, and $v_{i,jk}$. Since such derivatives can assume arbitrary values [2], we can enunciate the following theorem:

**Theorem** **2.** *The entropy inequality (17) is satisfied whatever the thermodynamic process is, if, and only if, the following thermodynamic restrictions hold:*

(18) $$\langle \frac{\partial s}{\partial \varepsilon_{,i}}\frac{\partial q_{k}}{\partial \varrho_{,j}}\rangle =0,$$

(19) $$\langle \frac{\partial s}{\partial \varepsilon_{,i}}\frac{\partial q_{k}}{\partial \varepsilon_{,j}}\rangle =0,$$

(20) $${\langle \frac{\partial s}{\partial \varepsilon_{,i}}T_{,kj}+\varrho^{2}\frac{\partial s}{\partial \varrho_{,i}}\delta_{,kj}\rangle }_{ij}=0,$$

(21) $$\langle \frac{\partial J_{i}}{\partial \varrho_{,j}}-\frac{\partial s}{\partial \varepsilon }\frac{\partial q_{i}}{\partial \varrho_{,j}}+\varrho v_{i}\frac{\partial s}{\partial \varrho_{,j}}\;-\varrho v_{j}\frac{\partial s}{\partial \varrho_{,i}}\rangle =0,$$

(22) $$\langle \frac{\partial J_{i}}{\partial \varepsilon_{,j}}-\frac{\partial s}{\partial \varepsilon }\frac{\partial q_{i}}{\partial \varepsilon_{,j}}+\varrho v_{i}\frac{\partial s}{\partial \varepsilon_{,j}}-\varrho v_{j}\frac{\partial s}{\partial \varepsilon_{,i}}\rangle =0,$$

(23) $$T_{ij}={\left(\frac{\partial s}{\partial \varepsilon }\right)}^{-1}\left(\varrho^{2}\frac{\partial s}{\partial \varrho }+\frac{\partial s}{\partial \varrho_{,k}}\varrho_{,k}\delta_{ij}+\frac{\partial s}{\partial \varrho_{,j}}\varrho_{,i}\right),$$

(24) $$\begin{array}{c} \;-\frac{\partial s}{\partial \varepsilon }\left(\frac{\partial q_{i}}{\partial \varrho }\varrho_{,i}+\frac{\partial q_{i}}{\partial \varepsilon }\varepsilon_{,i}\right)-\;\varrho^{2}\frac{\partial s}{\partial \varrho }v_{i,i}+\frac{\partial J_{i}}{\partial \varrho }\varrho_{,i}+\frac{\partial J_{i}}{\partial \varepsilon }\varepsilon_{,i}\geq 0. \end{array}$$

It is evident that the restrictions above do not prevent *s* to depend on the gradient of ϱ. The constitutive equation for the Cauchy stress in Equation (1), with α=γ=0, can be achieved by taking

(25) $$s=s_{0}\left(\varrho ,\varepsilon \right)+s_{1}\left(\varrho \right)\varrho_{,k}\varrho_{,k}+s_{2}\left(\varepsilon \right)\varrho_{,k}\varrho_{,k}$$

with

(26) $$\varrho^{2}\vartheta \frac{\partial s_{0}}{\partial \varrho }=-p,\;\;\;\;\;\;\;\;\frac{1}{2}\varrho^{2}\vartheta \frac{\partial s_{1}}{\partial \varrho }+s_{1}+s_{2}=\beta ,\;\;\;\;\;\;\;s_{1}+s_{2}=\delta ,$$

wherein the thermodynamic relation $\frac{\partial s}{\partial \varepsilon }=\frac{1}{\vartheta }$ has been used.

**Remark** **5.** *By relaxing the semi-linear approximation, the restrictions above would be more complex. However, our aim here is not to develop a complete thermodynamic model of second-grade Korteweg fluids, for which we refer the reader to *[2]*, but simply to show how the inclusion of the gradient extensions into the dissipation inequality ensures the thermodynamic compatibility.*

### 1.2. Second Law and Extended Procedures

Let *B* be a continuous body whose evolution in spacetime is governed by a system of balance laws. Here, and in the following, we assume that memory effects are negligible for *B*, so that the constitutive equations that characterize its material properties are functions defined on a local-in-time state space, and not on a space of histories. Our results do not cover the non-equilibrium history effects addressed by the celebrated theory of Coleman [6], but one should note that this is not the sole way of accounting for non-equilibrium effects. To this end, the presence of gradients in the state space allows one to represent inhomogeneities that are typical of non-equilibrium situations. For instance, inhomogeneity in the temperature produces a heat flux, which pushes the system out of equilibrium. According to such a point of view, in addition, the actions at distance are supposed to be weakly non-local, in such a way that the response of *B* in a point depends only on the value of the state functions and of their spatial gradients in a small neighborhood of that point. In this way, the evolution of *B* in space and time can be described by a system of partial differential equations, with unknown functions defined in $R^{3}\times \left[0,\;\infty \right]$. Finally, one should note that the Coleman approach to materials with memory relies on an additional physical assumption, the fading memory (see Section 5 in [6]), which makes the resulting system of integro-differential equations mathematically manageable. However, as proven by Fichera [13], such a hypothesis depends on the choice of a suitable function space and, for elastic materials, it becomes completely meaningless due to the impossibility of choosing, on the basis of pure physical considerations, an appropriate function space.

Thus, here we limit ourselves to consider a system of partial differential equations. It can be represented as follows,

(27) $$z_{\beta ,t}+z_{\beta ,j}v_{j}+\Phi_{k,k}^{\beta }=r_{\beta },\;\;\;\;\;\beta =1\ldots N,$$

with $v_{j}$ as the components of the velocity field on *B* entering the total time derivative, $\Phi_{k}^{\beta }$ as the components of the flux of $z_{\beta }$, and $r_{\beta }$ as the production of $z_{\beta }$ (for the sake of simplicity, we assume that the supplies are zero). The equations above can represent, for instance, the Grad hierarchical system of extended thermodynamics [14]. However, if the productions, $r_{\beta }$, are equal to zero, it can also represent the balances of mass, linear momentum, and energy of rational thermodynamics in the absence of source terms [15]. We suppose that the fluxes, $\Phi_{k}^{\beta }$, and the productions, $r_{\beta }$, depend on *N* unknown fields, $z_{\alpha }\left(x_{j},\;t\right)$, and on their spatial derivatives, $z_{\alpha ,j}\left(x_{j},\;t\right)$. Their constitutive equations must be assigned in accordance to constraint (4). Such a requirement, which is known as the dissipation (or entropy) principle, was clearly established for the first time by Coleman and Noll in 1963 [4], and later on by Coleman and Mizel [5]. Coleman established the same principle in dealing with materials with memory [6]. It is worth observing that even though the dissipation principle is a useful operative assumption it is not dictated by a general physical law. Thus, in principle, nothing prevents one from regarding the second law as a restriction on the thermodynamic processes by selecting, among the solutions of Equation (27), those that can actually occur in nature. In 1996, in order to decide the correct approach, Muschik and Ehrentraut [11] proposed the amendment to the second law mentioned above. It expresses the physical evidence that in a continuum body the entropy production is zero if, and only if, it is in thermodynamic equilibrium. Under the validity of the amendment, those authors proved that the second law of thermodynamics necessarily restricts the constitutive equations and not the thermodynamic processes. In 2022, Cimmelli and Rogolino, with the aim of formulating this important result within a geometric framework, generalized this result by including such an amendment in a more general formulation of second law of thermodynamics [16]. Finally, in 2023, Cimmelli further generalized the previous result by encompassing non-regular processes and shock-wave propagation [17]. It is worth noting that in [16,17] only classical (i.e., not extended) exploitation procedures have been considered. Thus, the aim of the present article is to reformulate the Muschik and Ehrentraut theorem in such a way as to include the extended exploitation procedures illustrated above.

The paper runs as follows.

In Section 2, we provide an overview of a new thermodynamic framework for non-equilibrium thermodynamics developped in [16]. In Section 3, we prove the Muschik and Ehrentraut theorem in the case of extended exploitation procedures. In Section 4, we apply an extended procedure to investigate the thermodynamic compatibility of the strain-gradient elasticity [18,19]. Finally, conclusions and future perspectives are illustrated in Section 5.

## 2. A Geometric Framework for Thermodynamics

In this section, we resume the geometric framework developed in [16] in order to formulate our main result. To this end, we start by providing some basic definitions already introduced in [16].

The *N*-dimensional vector space, $C_{t}$, structured as a finite-dimensional manifold, spanned by the solutions $z_{\alpha }\left(x_{j},\;t\right)$ of Equation (27), is called the space of the configurations at the instant *t*.

The disjoint union

(28) $$C=\underset{t\in [0,\;\infty ]}{⋃}\left\{t\right\}\times C_{t},$$

structured as a fiber bundle over [0,∞], is called the configuration bundle.

If $C_{t}=C\;\forall t$, then C has the topology of the Cartesian product,

(29) C=[0,∞]×C.

A thermodynamic process, π, of initial point $\tau_{0}$ and duration τ is a vector valued function, $\pi :t\in \left[\tau_{0},\;\tau_{0}+\tau \right]\subseteq \left[0,\;\infty \right]\to z_{\alpha }\left(x_{j},\;t\right)\in C$.

For $t_{0}\in ]\tau_{0},\;\tau_{0}+\tau ]$, a restricted thermodynamic process, *p*, of initial point $t_{0}$ and duration $\tau_{0}+\tau -t_{0}$ is a vector-valued function, $p:t\in \left[t_{0},\;\tau_{0}+\tau \right]\subseteq \left[0,\;\infty \right]\to z_{\alpha }\left(x_{j},\;t\right)\in C$. In order to solve system (27), suitable constitutive equations for the quantities $\Phi_{k}^{\alpha }$ and $r_{\alpha }$ must be assigned on a chosen state space.

The 4N-dimensional vector space,

(30) $$\Sigma_{t}=\left\{z_{\alpha }\left(x_{j},\;t\right),\;z_{\alpha ,j}\left(x_{j},\;t\right)\right\},$$

for any value of *t* is the state space at the instant *t*, while the disjoint union,

(31) $$S=\underset{t\in [0,\;\infty ]}{⋃}\left\{t\right\}\times \Sigma_{t},$$

is its thermodynamic bundle.

On $\Sigma_{t}$, the balance Equation (27) and the local form of the second law of thermodynamics (local entropy inequality) take the forms

(32) $$z_{\alpha ,t}+z_{\alpha ,k}v_{k}+\frac{\partial \Phi_{j}^{\alpha }}{\partial z_{\beta }}z_{\beta ,j}+\frac{\partial \Phi_{k}^{\alpha }}{\partial z_{\beta ,k}}z_{\beta ,kj}=r_{\alpha },$$

(33) $$\sigma^{\left(s\right)}=\varrho \frac{\partial s}{\partial z_{\alpha }}z_{\alpha ,t}+\varrho \frac{\partial s}{\partial z_{\alpha ,j}}z_{\alpha ,jt}+\varrho \frac{\partial s}{\partial z_{\alpha }}z_{\alpha ,j}v_{j}+\varrho \frac{\partial s}{\partial z_{\alpha ,k}}z_{\alpha ,kj}v_{j}+\frac{\partial J_{k}}{\partial z_{\alpha }}z_{\alpha ,k}+\frac{\partial J_{k}}{\partial z_{\alpha ,j}}z_{\alpha ,jk}\geq 0.$$

Equation (27) can be rewritten in compact form as

(34) $$A_{\beta \alpha }\left(S\right)y_{\alpha }=C_{\beta }\left(S\right),$$

with the 10N×1 column vector function

(35) $$y_{\alpha }\equiv {\left(z_{\alpha ,t},\;\;z_{\alpha ,jt},\;\;z_{\alpha ,kj}\right)}^{T},$$

the N×1 column vector

(36) $$C_{\beta }\equiv r_{\beta }-z_{\beta ,j}v_{j}-\frac{\partial \Phi_{j}^{\beta }}{\partial z_{\alpha }}z_{\alpha ,j},\;\;\;\;\beta =1\ldots N,$$

and the N×10N matrix

(37) $$A_{\beta \alpha }\equiv \left[\delta_{\beta \alpha },\;\;\;\;\frac{\partial \Phi_{j}^{\beta }}{\partial z_{\alpha ,k}}\right]\;\;\;\;\;\left(j,\;k=1,2,3\right),$$

where $C_{\beta }$ and $A_{\beta \alpha }$ depend on the elements of S.

Analogously, inequality (33) can be rearranged as follows,

(38) $$B_{\alpha }\left(S\right)y_{\alpha }\geq D\left(S\right),$$

with the 10N×1 column vector

(39) $$B_{\alpha }\left(S\right)\equiv {\left(\rho \frac{\partial s}{\partial z_{\alpha }},\;\;\;\;\rho \frac{\partial s}{\partial z_{\alpha ,j}},\;\;\;\;\left(\rho \frac{\partial s}{\partial z_{\alpha ,k}}v_{i}+\frac{\partial J_{i}}{\partial z_{\alpha ,k}}\right)\right)}^{\;T}$$

and the scalar function

(40) $$D\left(S\right)\equiv -\rho \frac{\partial s}{\partial z_{\alpha }}z_{\alpha ,j}v_{j}-\frac{\partial J_{i}}{\partial z_{\alpha }}z_{\alpha ,i}.$$

The local-in-time 10N-dimensional vector space,

(41) $$H_{t}=\left\{z_{\alpha ,t},\;z_{\alpha ,mt},\;z_{\alpha ,jk}\right\},$$

and the fiber bundle,

(42) $$H=\underset{t\in [0,\;\infty ]}{⋃}\left\{t\right\}\times H_{t},$$

represent the space of the higher derivatives that, at the instant *t*, satisfy balance Equation (34) and its fiber bundle, respectively. Moreover, the 6N-dimensional equilibrium subspace of $H_{t}$ and its fiber bundle are provided by

(43) $$E_{t}=\left\{z_{\alpha ,jk}\right\}$$

and

(44) $$E=\underset{t\in [0,\;\infty ]}{⋃}\left\{t\right\}\times E_{t}.$$

The local-in-time 10N-dimensional vector space at time *t*,

(45) $$W_{t}=\left\{z_{\alpha ,t},\;z_{\alpha ,mt},\;z_{\alpha ,jk}\right\},$$

and the fiber bundle,

(46) $$W=\underset{t\in [0,\;\infty ]}{⋃}\left\{t\right\}\times W_{t},$$

represent the vector space of the higher derivatives that, at the instant *t*, satisfy the entropy inequality Equation (33) and its fiber bundle, respectively. Moreover, the 6N-dimensional equilibrium subspace of $W_{t}$ and its fiber bundle are provided by

(47) $${\hat{E}}_{t}=\left\{z_{\alpha ,jk}\right\}$$

and

(48) $$\hat{E}=\underset{t\in [0,\;\infty ]}{⋃}\left\{t\right\}\times E_{t}.$$

Let us now consider a fixed point, $P_{0}\in B$, and a fixed instant of time, $t_{0}\in \left[\tau_{0},\;\tau_{0}+\tau \right]$. Whatever $t_{0}$ is, it can always be considered as the initial time of a restricted process of duration $\tau_{0}+\tau -t_{0}$. When evaluated in $\left(P_{0},\;t_{0}\right)$, balance Equation (27) and entropy inequality (33) transform into the algebraic relations

(49) $$A_{\beta \alpha }\left(\Sigma_{0}\right)y_{\alpha }=C_{\beta }\left(\Sigma_{0}\right),$$

(50) $$\sigma^{\left(s\right)}\left(\Sigma_{0}\right)=B_{\alpha }\left(\Sigma_{0}\right)y_{\alpha }-D\left(\Sigma_{0}\right)\geq 0,$$

where $\Sigma_{0}$ is the vector space $\Sigma_{t}\left(P_{0},\;t_{0}\right)$. In the following, the spaces $H_{0}=H_{t}\left(P_{0},\;t_{0}\right)$ and $E_{0}=E_{t}\left(P_{0},\;t_{0}\right)$ will also be considered. From now on, we pursue our analysis under the hypothesis that *B* occupies the whole space. Then, for arbitrary $t_{0}\in \left[\tau_{0},\tau_{0}+\tau \right]$ we consider the restricted process, *p*, of initial instant, $t_{0}$, and duration, $\tau_{0}+\tau -t_{0}$, and suppose that it corresponds to the solution of the Cauchy problem for the system (27) with the initial conditions

(51) $$z_{\alpha }\left(x_{j},\;t_{0}\right)=z_{\alpha \;0}\left(x_{j}\right),\;\;\;\;\forall P\in C.$$

The problem concerning (27) and (51) in general is very difficult to solve, so finding a solution for it and verifying ex post if it also satisfies (33) is not easy. For this reason, in 1963 Coleman and Noll [4] postulated the constitutive principle referred to in Section 1, ref. [20]. Therefore, it is important to investigate if the Coleman and Noll postulate is a consequence of a general physical law or if it is an arbitrary, although very useful, assumption [11].

To this end, we observe that, since all the elements of Equation (27) have been substituted into the entropy inequality, we do not have other equations allowing us to determine the value of the higher derivatives in $\left(P_{0},\;t_{0}\right)$. Of course, by spatial derivation of the initial conditions (51), we obtain

(52) $$z_{\alpha ,jk}\left(x_{j},\;t_{0}\right)=z_{\alpha \;0,jk}\left(x_{j}\right),$$

which, once evaluated in $P_{0}$, provides 6N components of $y_{\alpha }$. However, since the initial conditions (51) can be assigned arbitrarily, the 6N quantities determined by Equation (52) can assume arbitrary values. Thus, the 10N components of $y_{\alpha }$ are completely arbitrary. Then, it is not guaranteed that the inequality (50) is satisfied, whatever $y_{\alpha }$ is. Such an observation suggests the following definitions.

A vector $y_{\alpha }\in H_{0}$ is said to be the following:

- real, if $\sigma^{\left(s\right)}\left(y_{\alpha }\right)\left(P_{0},\;t_{0}\right)>0$;
- ideal, if $\sigma^{\left(s\right)}\left(y_{\alpha }\right)\left(P_{0},\;t_{0}\right)=0$;
- over-ideal, if $\sigma^{\left(s\right)}\left(y_{\alpha }\right)\left(P_{0},\;t_{0}\right)<0$.

The definitions above allow us to establish the following assumption, whose experimental meaning is straightforward.

**Assumption** **1.** **Second law of thermodynamics (local formulation)** *. The local space of the higher derivatives, $W_{0}$, does not contain over-ideal vectors. Moreover, a vector, $y_{\alpha }\in W_{0}$, is ideal if, and only if, $\left(P_{0},\;t_{0}\right)$ is in thermodynamic equilibrium.*

**Remark** **6.** *We note that the local formulation of the second law of thermodynamics prohibits over-ideal vectors from being in $W_{0}$ but does not prevent them from being in $H_{0}$.*

Let γ be the curve representative in C of an arbitrary thermodynamic process of initial instant $t_{0}$ and duration $\tau_{0}+\tau -t_{0}$. The process, *p*, is said to be as follows:

- reversible, if in any point of γ$\sigma^{\left(s\right)}\left(y_{\alpha }\right)\left(P_{0},\;t_{0}\right)=0$;
- over-reversible, if there exists a point of γ in which $\sigma^{\left(s\right)}\left(y_{\alpha }\right)\left(P_{0},\;t_{0}\right)<0$;
- irreversible, if *p* is not over-reversible and there exists a point of γ in which $\sigma^{\left(s\right)}\left(y_{\alpha }\right)\left(P_{0},\;t_{0}\right)>0$.

The definitions above allow one to enunciate the following assumption.

**Assumption** **2.** **Second law of thermodynamics (global formulation)** *. Over-reversible processes do not occur in nature. Moreover, a thermodynamic process is reversible if, and only if, any point, P∈B, at any instant, t, is in thermodynamic equilibrium.*

Owing to Assumptions 1 and 2, the following results have been proven in [16]:

**Theorem** **3.** *If Assumption 1 is true, then, $H_{0}$ = $W_{0}$.*

**Corollary** **1.** *H = W.*

**Theorem** **4.** *If Assumptions 1 and 2 are true, then the second law restricts the constitutive equations and not the thermodynamic processes.*

## 3. The Muschik and Ehrentraut Theorem for Extended Procedures

For the purposes clarified in Section 1, we need to calculate the spatial differential consequences of balance Equation (27), namely, their gradient extensions. They read

(53) $$z_{\alpha ,mt}+z_{\alpha ,km}v_{k}+z_{\alpha ,k}v_{k,m}+\frac{\partial^{2}\Phi_{j}^{\alpha }}{\partial z_{\gamma }\partial z_{\beta }}z_{\beta ,j}z_{\gamma ,m}+\frac{\partial^{2}\Phi_{j}^{\alpha }}{\partial z_{\gamma ,k}\partial z_{\beta }}z_{\beta ,j}z_{\gamma ,km}+\frac{\partial \Phi_{j}^{\alpha }}{\partial z_{\beta }}z_{\beta ,jm}+$$

$$\frac{\partial^{2}\Phi_{j}^{\alpha }}{\partial z_{\gamma }\partial z_{\beta ,k}}z_{\beta ,kj}z_{\gamma ,m}+\frac{\partial^{2}\Phi_{j}^{\alpha }}{\partial z_{\gamma ,l}\partial z_{\beta ,k}}z_{\beta ,kj}z_{\gamma ,lm}+\frac{\partial \Phi_{j}^{\alpha }}{\partial z_{\beta ,k}}z_{\beta ,kjm}=\frac{\partial r_{\alpha }}{\partial z_{\gamma }}z_{\gamma ,m}+\frac{\partial r_{\alpha }}{\partial z_{\gamma ,l}}z_{\gamma ,lm}.$$

In Equation (53), we may individuate the 10N highest derivatives, $\left\{z_{\alpha ,jkm}\right\}$, which are the highest spatial derivatives of the unknown fields. The (local-in-time) space of the highest derivatives and its fiber bundle are provided by

(54) $$U_{t}=\left\{z_{\alpha ,jkm}\right\}$$

and

(55) $$U=\underset{t\in [0,\;\infty ]}{⋃}\left\{t\right\}\times U_{t},$$

respectively.

It is worth observing that inequality (33) holds for arbitrary thermodynamic processes, so that it does not contain any information on the type of process ruled by Equation (27), i.e., on the evolution of the independent thermodynamic variables, $\left\{z_{\alpha }\left(x_{j},\;t\right),\;z_{\alpha ,j}\left(x_{j},\;t\right)\right\}$. Thus, it is necessary to substitute Equations (27) and (53) into inequality (33). We then obtain

(56) $$\varrho \frac{\partial s}{\partial z_{\alpha }}\left[-z_{\alpha ,k}v_{k}-\frac{\partial \Phi_{j}^{\alpha }}{\partial z_{\beta }}z_{\beta ,j}-\frac{\partial \Phi_{k}^{\alpha }}{\partial z_{\beta ,k}}z_{\beta ,kj}+r_{\alpha }\right]+$$

$$+\varrho \frac{\partial s}{\partial z_{\alpha ,m}}\left[-z_{\alpha ,km}v_{k}-z_{\alpha ,k}v_{k,m}-\frac{\partial^{2}\Phi_{j}^{\alpha }}{\partial z_{\gamma }\partial z_{\beta }}z_{\beta ,j}z_{\gamma ,m}-\frac{\partial^{2}\Phi_{j}^{\alpha }}{\partial z_{\gamma ,k}\partial z_{\beta }}z_{\beta ,j}z_{\gamma ,km}-\frac{\partial \Phi_{j}^{\alpha }}{\partial z_{\beta }}z_{\beta ,jm}\right]$$

$$+\varrho \frac{\partial s}{\partial z_{\alpha ,m}}\left[-\frac{\partial^{2}\Phi_{j}^{\alpha }}{\partial z_{\gamma }\partial z_{\beta ,k}}z_{\beta ,kj}z_{\gamma ,m}-\frac{\partial^{2}\Phi_{j}^{\alpha }}{\partial z_{\gamma ,l}\partial z_{\beta ,k}}z_{\beta ,kj}z_{\gamma ,lm}-\frac{\partial \Phi_{j}^{\alpha }}{\partial z_{\beta ,k}}z_{\beta ,kjm}+\frac{\partial r_{\alpha }}{\partial z_{\gamma }}z_{\gamma ,m}+\frac{\partial r_{\alpha }}{\partial z_{\gamma ,l}}z_{\gamma ,lm}\right]+$$

$$+\varrho \frac{\partial s}{\partial z_{\alpha }}z_{\alpha ,j}v_{j}+\varrho \frac{\partial s}{\partial z_{\alpha ,k}}z_{\alpha ,kj}v_{j}+\frac{\partial J_{k}}{\partial z_{\alpha }}z_{\alpha ,k}+\frac{\partial J_{k}}{\partial z_{\alpha ,j}}z_{\alpha ,jk}\geq 0.$$

The inequality above can be rewritten as follows:

(57) $$-\varrho \frac{\partial s}{\partial z_{\alpha }}\frac{\partial \Phi_{k}^{\alpha }}{\partial z_{\beta ,k}}z_{\beta ,kj}+\varrho \frac{\partial s}{\partial z_{\alpha ,k}}z_{\alpha ,kj}v_{j}+\frac{\partial J_{k}}{\partial z_{\alpha ,j}}z_{\alpha ,jk}$$

$$-\varrho \frac{\partial s}{\partial z_{\alpha ,m}}\left[z_{\alpha ,km}v_{k}+\frac{\partial^{2}\Phi_{j}^{\alpha }}{\partial z_{\gamma ,k}\partial z_{\beta }}z_{\beta ,j}z_{\gamma ,km}+\frac{\partial \Phi_{j}^{\alpha }}{\partial z_{\beta }}z_{\beta ,jm}\right]$$

$$-\varrho \frac{\partial s}{\partial z_{\alpha ,m}}\left[\frac{\partial^{2}\Phi_{j}^{\alpha }}{\partial z_{\gamma }\partial z_{\beta ,k}}z_{\beta ,kj}z_{\gamma ,m}+\frac{\partial \Phi_{j}^{\alpha }}{\partial z_{\beta ,k}}z_{\beta ,kjm}+\frac{\partial r_{\alpha }}{\partial z_{\gamma ,l}}z_{\gamma ,lm}\right]$$

$$-\varrho \frac{\partial s}{\partial z_{\alpha ,m}}\frac{\partial^{2}\Phi_{j}^{\alpha }}{\partial z_{\gamma ,l}\partial z_{\beta ,k}}z_{\beta ,kj}z_{\gamma ,lm}$$

$$\varrho \frac{\partial s}{\partial z_{\alpha }}\left[-z_{\alpha ,k}v_{k}-\frac{\partial \Phi_{j}^{\alpha }}{\partial z_{\beta }}z_{\beta ,j}+r_{\alpha }\right]-\varrho \frac{\partial s}{\partial z_{\alpha ,j}}z_{\alpha ,k}v_{k,j}-\varrho \frac{\partial s}{\partial z_{\alpha ,m}}\frac{\partial^{2}\Phi_{j}^{\alpha }}{\partial z_{\gamma }\partial z_{\beta }}z_{\beta ,j}z_{\gamma ,m}$$

$$+\varrho \frac{\partial s}{\partial z_{\alpha }}z_{\alpha ,j}v_{j}+\varrho \frac{\partial s}{\partial z_{\alpha ,j}}\frac{\partial r_{\alpha }}{\partial z_{\gamma }}z_{\gamma ,j}+\varrho \frac{\partial s}{\partial z_{\alpha }}z_{\alpha ,j}v_{j}+\frac{\partial J_{k}}{\partial z_{\alpha }}z_{\alpha ,k}\geq 0.$$

In inequality (57), we may individuate the 10N highest derivatives, $\left\{z_{\alpha ,jkm}\right\}$, which are the highest spatial derivatives of the unknown fields entering the generalized entropy inequality. The local-in-time space of such highest derivatives and their fiber bundles are provided by

(58) $${\hat{U}}_{t}=\left\{z_{\alpha ,jkm}\right\}$$

and

(59) $$\hat{U}=\cup_{t\in [0,\;\infty ]}\left\{t\right\}\times {\hat{U}}_{t},$$

respectively. We note that the time derivatives have now been eliminated from the entropy inequality so that all the higher derivatives therein are elements of ${\hat{E}}_{t}$.

The first three lines in Equation (57) are constituted by the terms that are linear in the elements ${\hat{e}}_{\alpha }\in {\hat{E}}_{t}$ and in the elements ${\hat{u}}_{\alpha }\in {\hat{U}}_{t}$, the fourth line is quadratic in the elements ${\hat{e}}_{\alpha }\in {\hat{E}}_{t}$, and the last two lines contain only terms that are defined on the state space. Thus, it can be written as

(60) $$B_{\alpha }\left(\Sigma_{t}\right){\hat{e}}_{\alpha }+F_{\alpha }\left(\Sigma_{t}\right){\hat{u}}_{\alpha }+C_{\alpha \beta }\left(\Sigma_{t}\right){\hat{e}}_{\alpha }{\hat{e}}_{\beta }\geq D\left(\Sigma_{t}\right),$$

with ${\hat{e}}_{\alpha }$ and ${\hat{u}}_{\alpha }$ as elements of ${\hat{E}}_{t}$ and ${\hat{U}}_{t}$, respectively, $B_{\alpha }$ a 6N×1 column vector, $F_{\alpha }$ a 10N×1 column vector, $C_{\alpha \beta }$ a 6N×6N matrix, and *D* a scalar. Moreover, when evaluated in $\left(P_{0},\;t_{0}\right)$, it yields

(61) $$B_{\alpha }\left(\Sigma_{0}\right){\hat{e}}_{\alpha }+F_{\alpha }\left(\Sigma_{0}\right){\hat{u}}_{\alpha }+C_{\alpha \beta }\left(\Sigma_{0}\right){\hat{e}}_{\alpha }{\hat{e}}_{\beta }\geq D\left(\Sigma_{0}\right),$$

with ${\hat{e}}_{\alpha }\in {\hat{E}}_{0}$ and ${\hat{u}}_{\alpha }\in {\hat{U}}_{0}$.

Theorems 3 and 4 cannot be considered as valid for the generalized procedures considered here, since inequality (57) contains elements that are not present in inequality (33), belonging to vector spaces that are different from $H_{0}$ and $W_{0}$. In particular, the linear terms in the 10N highest derivatives, which can be obtained by a further spatial derivative of the initial conditions (51), are then completely arbitrary, and the quadratic terms in the higher derivatives, also completely arbitrary, do not appear in inequality (33). Thus, we define the vector space, $V_{t}=E_{t}\cup U_{t}$, which contains the set of the higher and highest derivatives satisfying the gradient extensions (53), and the vector space ${\hat{V}}_{t}={\hat{E}}_{t}\cup {\hat{U}}_{t}$, which contains the set of the higher and highest derivatives satisfying the entropy inequality (56). Their fiber bundles are, respectively, $V=\cup_{t\in [0,\;\infty ]}\left\{t\right\}\times V_{t}$ and $\hat{V}=\cup_{t\in [0,\;\infty ]}\left\{t\right\}\times {\hat{V}}_{t}$. Moreover, $V_{0}$ and ${\hat{V}}_{0}$ denote such spaces evaluated in $\left(P_{0},\;t_{0}\right)$. Our aim is to investigate if ${\hat{V}}_{0}=V_{0}$ or ${\hat{V}}_{0}\subset V_{0}$. To this end, we define the subspace ${\hat{V}}_{0\;id}$ of ${\hat{V}}_{0}$ constituted by the vectors ${\hat{v}}_{\alpha }=\left\{{\hat{e}}_{\alpha },\;{\hat{u}}_{\alpha }\right\}\in {\hat{V}}_{0}$, such that $B_{\alpha }\left(\Sigma_{0}\right){\hat{e}}_{\alpha }+F_{\alpha }\left(\Sigma_{0}\right){\hat{u}}_{\alpha }+C_{\alpha \beta }\left(\Sigma_{0}\right){\hat{e}}_{\alpha }{\hat{e}}_{\beta }=D\left(\Sigma_{0}\right)$.

A vector, $v_{\alpha }=\left\{e_{\alpha },\;u_{\alpha }\right\}\in V_{0}$, is said to be as follows:

- admissible, if $v_{\alpha }\in {\hat{V}}_{0}-{\hat{V}}_{0\;id}$;
- ideally admissible, if $v_{\alpha }\in {\hat{V}}_{0\;id}$;
- not-admissible, if $v_{\alpha }\in V_{0}-{\hat{V}}_{0}$.

We can now prove the following proposition:

**Theorem** **5.** *Let B be a body, and let the couple $\left(P_{0},\;t_{0}\right)$ represent an arbitrary point of B at an arbitrary instant $t_{0}\in \left[\tau_{0},\;\tau_{0}+\tau \right]$. Then, $V_{0}={\hat{V}}_{0}$.*

**Proof.**  We start by observing that in a point $\left(P_{0},\;t_{0}\right)$ the vector space, $V_{0}$, cannot contain both admissible and ideally admissible vectors, nor both not-admissible and ideally admissible vectors, otherwise the point should be in both thermodynamic equilibrium and outside of thermodynamic equilibrium. Thus, in $V_{0}$ there are either only ideally admissible vectors (equilibrium), or only admissible and not-admissible vectors (out of equilibrium).

Let $y_{\alpha }^{1}$ be a real vector and $y_{\alpha }^{2}$ an over-ideal vector of $H_{0}$, and let us suppose that $y_{\alpha }^{1}$ corresponds to an admissible vector in $V_{0}$ and $y_{\alpha }^{2}$ corresponds to a not-admissible vector in $V_{0}$. Let us consider the linear combination $y_{\alpha }^{3}=\lambda y_{\alpha }^{1}+\left(1-\lambda \right)y_{\alpha }^{2}$, with λ∈]0,1[. Since $y_{\alpha }^{1}$ and $y_{\alpha }^{2}$ are in $H_{0}$, they satisfy the following equations:

(62) $$A_{\beta \alpha }\left(\Sigma_{0}\right)y_{\alpha }^{1}=C_{\beta }\left(\Sigma_{0}\right),$$

(63) $$A_{\beta \alpha }\left(\Sigma_{0}\right)y_{\alpha }^{2}=C_{\beta }\left(\Sigma_{0}\right).$$

The combination of Equation (62) multiplied by λ and Equation (63) multiplied by (1−λ) leads to

(64) $$A_{\beta \alpha }\left(\Sigma_{0}\right)y_{\alpha }^{3}=C_{\beta }\left(\Sigma_{0}\right),$$

namely, $y_{\alpha }^{3}$ is also in $H_{0}$. Let $v_{\alpha }^{1}=\left(e_{\alpha }^{1},\;u_{\alpha }^{1}\right)$ be the admissible vector corresponding to element $y_{\alpha }^{1}$ and $v_{\alpha }^{2}=\left(e_{\alpha }^{2},\;u_{\alpha }^{2}\right)$ be the not-admissible vector, corresponding to element $y_{\alpha }^{2}$. It is fundamental to observe that, with $y_{\alpha }^{1}$ being real and $y_{\alpha }^{2}$ being over-ideal, as a consequence of the local formulation of the second law of thermodynamics point $\left(P_{0},\;t_{0}\right)$ is not in thermodynamic equilibrium.

Consider now the linear combination $v_{\alpha }=\lambda v_{\alpha }^{1}+\left(1-\lambda \right)v_{\alpha }^{2}$, which corresponds to the solution $y_{\alpha }^{3}$ of Equation (64).

The entropy production corresponding to $v_{\alpha }$ is

(65) $$\sigma \left(v_{\alpha },\lambda \right)=\lambda \left[B_{\alpha }\left(\Sigma_{0}\right)e_{\alpha }^{1}+F_{\alpha }\left(\Sigma_{0}\right)u_{\alpha }^{1}+C_{\alpha \beta }\left(\Sigma_{0}\right)e_{\alpha }^{1}e_{\beta }^{1}-D\left(\Sigma_{0}\right)\right]+$$

$$\left(1-\lambda \right)[B_{\alpha }\left(\Sigma_{0}\right)e_{\alpha }^{2}+F_{\alpha }\left(\Sigma_{0}\right)u_{\alpha }^{2}+C_{\alpha \beta }\left(\Sigma_{0}\right)e_{\alpha }^{2}e_{\beta }^{2}-D\left(\Sigma_{0}\right)].$$

We note that the right-hand side of Equation (65) is a continuous function of λ in the interval [0,1]. On the other hand, with $\left\{e_{\alpha }^{2},\;u_{\alpha }^{2}\right\}$ being not-admissible and $\left\{e_{\alpha }^{1},\;u_{\alpha }^{1}\right\}$ being admissible, we have

(66) $$\sigma \left(v_{\alpha },\lambda =0\right)=\left[B_{\alpha }\left(\Sigma_{0}\right)e_{\alpha }^{2}+F_{\alpha }\left(\Sigma_{0}\right)u_{\alpha }^{2}+C_{\alpha \beta }\left(\Sigma_{0}\right)e_{\alpha }^{2}e_{\beta }^{2}-D\left(\Sigma_{0}\right)\right]<0$$

and

(67) $$\sigma \left(v_{\alpha },\lambda =1\right)=\left[B_{\alpha }\left(\Sigma_{0}\right)e_{\alpha }^{1}+F_{\alpha }\left(\Sigma_{0}\right)u_{\alpha }^{1}+C_{\alpha \beta }\left(\Sigma_{0}\right)e_{\alpha }^{1}e_{\beta }^{1}-D\left(\Sigma_{0}\right)\right]>0.$$

Hence, with $\sigma (v_{\alpha }\;\lambda )$ being a continuous function of λ in the interval [0,1], there exists a value $\bar{\lambda }\in ]0,\;1[$, such that $\sigma (v_{\alpha },\bar{\lambda })=0$. Thus, in $V_{0}$ there exists an ideally admissible vector, ${\bar{v}}_{\alpha }=\bar{\lambda }v_{\alpha }^{1}+\left(1-\bar{\lambda }\right)v_{\alpha }^{2}$, which contradicts the fact that $\left(P_{0},\;t_{0}\right)$ is out of equilibrium. Then, $V_{0}$ cannot contain both admissible and not-admissible vectors, i.e., the vectors of $V_{0}$ are either all admissible or all not-admissible. On the other hand, since the point $\left(P_{0},\;t_{0}\right)$ is arbitrary, if all the vectors in $V_{0}$ are not-admissible, no gradient extension of the solution of the balance laws (27) is compatible with the second law of thermodynamics. However, such gradient extensions enter the process-direction vectors $y_{\alpha }\in H_{0}$, and then such vectors are all over-ideal. Since this contradicts the local formulation of the second law of thermodynamics (Assumption 1), we must conclude that all the vectors in $V_{0}$ are admissible, i.e., $V_{0}={\hat{V}}_{0}$. □

**Remark** **7.** *With $\left(P_{0},\;t_{0}\right)$ being arbitrary, if all the process-direction vectors, $y_{\alpha }\in H_{0}$, are over-ideal, then all the thermodynamic processes are over-reversible, against the global formulation of the second law of thermodynamics (Assumption 2).*

**Corollary** **2.** *$\hat{V}$ = V.*

**Proof.**  This statement immediately follows the arbitrariness of the initial instant, $t_{0}$, and of the point $P_{0}$. □

**Theorem** **6.** *Inequality (57) restricts the constitutive equations and not the thermodynamic processes.*

**Proof.**  As a consequence of Theorem 5 and Remark 7, if $z_{\alpha }\left(x_{j},\;t\right)$ is a solution of the balance laws (27), the substitution of its gradient extension into the entropy inequality does not generate not-admissible vectors and, as a consequence, over-reversible processes that must be excluded by the second law of thermodynamics. As in the classical case investigated in [11,16], all the thermodynamic processes can only be either irreversible or reversible, but not over-reversible. On the other hand, given the state space, only for suitable forms of $\Phi_{k}^{\beta }$, $r_{\beta }$, *s*, and $J_{k}$ is the entropy production non-negative. Then, the role of the second law is only to select such forms. □

**Remark** **8.** *Regarding the consequences of inequality (61) on the constitutive equations, the following theorem has been proven in *[21]*:*

**Theorem** **7.** *Inequality (61) is satisfied by arbitrary vectors ${\hat{v}}_{\alpha }\in {\hat{V}}_{0}$ if, and only if,*

(68) $$B_{\alpha }\left(\Sigma_{0}\right)=0,\;\;F_{\alpha }\left(\Sigma_{0}\right)=0,\;\;D\left(\Sigma_{0}\right)>0$$

*and $C_{\alpha \beta }\left(\Sigma_{0}\right)$ is positive semidefinite.*

## 4. Strain Gradient Elasticity

When the dimensions of an elastic structure become comparable to the size of its material micro-structure, size effects and micro-structural effects manifest themselves at the macroscopic scale. Typical examples of such phenomena are those arising in nanostructured materials. Since the classical theory of elasticity is not capable of describing such behavior, a generalized constitutive theory, correlating the micro-structure with the macro-structure, is necessary.

Mindlin, in two celebrated papers [18,19], proposed two enhanced strain gradient elastic theories to describe the linear elastic behavior of isotropic materials with micro-structural effects. To achieve that task, he introduced a potential energy density depending not only on the strain but also on the gradient of the strain. From a thermodynamic point of view, such a dependency is difficult to achieve, because the classical Coleman–Noll procedure leads to the conclusion that the thermodynamic potentials, and hence the stress tensor, cannot depend on the spatial derivatives of the unknown fields. An efficient way to achieve such a compatibility is to modify the local balances of linear momentum and energy (first law of thermodynamics) through the introduction of a hyperstress tensor. The local balance of entropy (second law of thermodynamics) is also modified, by introducing a new thermal variable that reduces to the absolute temperature in the absence of micro-structural effects [22,23,24,25,26].

Our aim here is to show that, if the entropy inequality is exploited through the procedure illustrated in Section 1, then no modification is necessary and the compatibility can be proven directly by the classical entropy inequality. For the sake of simplicity, we limit ourselves to consider the one-dimensional case. Then, for a thermoelastic solid undergoing small deformations, the classical balances of linear momentum and energy, in the absence of body forces and heat sources, read

(69a) $$\begin{array}{cc} \; & \varrho \ddot{u}-T_{,x}=0, \end{array}$$

(69b) $$\begin{array}{cc} \; & \varrho \dot{\varepsilon }+q_{,x}-T\dot{E}=0, \end{array}$$

wherein ϱ is the mass density, *u* the displacement, *T* the stress tensor, ε the specific internal energy, $E=u_{,x}$ the strain tensor, and *q* the heat flux. Moreover, according to the fundamental tenets of extended irreversible thermodynamics (EIT) [27], we assign a balance law for the heat flux, namely,

(70) $$\tau \dot{q}+q=-\bar{\kappa }\varepsilon_{,x}-\Lambda E_{,x},$$

wherein $\bar{\kappa }=\kappa /c$, with κ being the thermal conductivity and *c* the specific heat. Finally, Λ is a thermophysical parameter having the dimension of a heat flux times a length.

Equation (70) generalizes the classical Maxwell–Cattaneo equation [28] by the presence of an additional heat flux due to the inhomogeneity of the strain. Also in this case, one can observe that, indeed, the classical Maxwell–Cattaneo equation,

(71) $$\tau \dot{q}+q=-\bar{\kappa }\varepsilon_{,x},$$

is a special case of the Volterra–Boltzmann integral for the history dependence of heat flux, namely, of the more general constitutive equation [29]:

(72) $$q=-\int_{0}^{\infty }a\left(s\right)g\left(t-s\right)ds.$$

When $g=\varepsilon_{,x}$, Equation (72) yields (71) under the hypothesis $a\left(s\right)=\left(\bar{\kappa }/\tau \right)\exp^{-s/\tau }$ [29]. As already observed in Section 1, in the present paper we restrict our study to systems of partial differential equations, and then we postulate Equation (70) as an extension of (71).

Thus, in order to close the system (69a)–(70), we must assign a constitutive equation for the stress tensor. We suppose that it is defined on the following weakly non-local state space:

(73) $$\Sigma =\left\{\varepsilon ,\;E,\;q,\;\varepsilon_{,x},\;E_{,x},\;q_{,x}\right\}.$$

We do not include $\dot{E}$ in Σ, as we did in [4,5], since we are interested only to explore the consequences of spatial weak non-locality and not those of viscosity on the constitutive equations.

We assume that *T* can be represented as the sum of the classical stress tensor of homogeneous and isotropic linear thermoelastic solids plus an extra-stress, which may also depend on the strain gradient, namely,

(74) $$T=\left(\lambda +2\mu \right)E-\bar{b}\varepsilon +\hat{T}\left(\varepsilon ,q,E,\varepsilon_{,x},E_{,x},\;q_{,x}\right),$$

with $\bar{b}=\left(3\lambda +2\mu \right)\alpha /c$ and α as the coefficient of thermal expansion. The coupling of Equations (69b) and (74) yields

(75) $$\varrho \dot{\varepsilon }+q_{,x}-\left(\lambda +2\mu \right)E\dot{E}+\bar{b}\varepsilon \dot{E}-\hat{T}\dot{E}=0.$$

In order to apply the procedure illustrated in Section 1, we need to calculate the gradient extension of Equations (70) and (75). Therefore, we obtain

(76a) $$\begin{array}{cc} \; & \tau {\dot{q}}_{,x}+q_{,x}=-\bar{\kappa }\varepsilon_{,xx}-\Lambda E_{,xx},\\ \; & \varrho {\dot{\varepsilon }}_{,x}+q_{,xx}+\left[-\left(\lambda +2\mu \right)E+\bar{b}\varepsilon -\hat{T}\right]{\dot{E}}_{,x}\;+ \end{array}$$

(76b) $$\begin{array}{cc} \; & \left[-\left(\lambda +2\mu \right)E_{,x}+\bar{b}\varepsilon_{,x}-\frac{\partial \hat{T}}{\partial \varepsilon }\varepsilon_{,x}-\frac{\partial \hat{T}}{\partial q}q_{,x}-\frac{\partial \hat{T}}{\partial E}E_{,x}-{\frac{\partial \hat{T}}{\partial \varepsilon }}_{,x}\varepsilon_{,xx}-\frac{\partial \hat{T}}{\partial q_{,x}}q_{,xx}-{\frac{\partial \hat{T}}{\partial E}}_{,x}E_{,xx}\right]\dot{E}=0. \end{array}$$

Balance Equation (70) and constitutive Equation (74) must satisfy the second law of thermodynamics, i.e., the local balance of entropy, which reads

(77) $$\varrho \dot{s}+J_{x}\geq 0,$$

with *s* as the specific entropy and *J* as the entropy flux. For these quantities, we must also assign a constitutive equation. The above inequality on the state space takes the form

(78) $$\begin{array}{c} \;\varrho \left(\frac{\partial s}{\partial \varepsilon }\dot{\varepsilon }+\frac{\partial s}{\partial q}\dot{q}+\frac{\partial s}{\partial E}\dot{E}+\frac{\partial s}{\partial \varepsilon_{,x}}{\dot{\varepsilon }}_{,x}+\frac{\partial s}{\partial q_{,x}}{\dot{q}}_{,x}+\frac{\partial s}{\partial E_{,x}}{\dot{E}}_{,x}\right)\\ \;+\frac{\partial J}{\partial \varepsilon }\varepsilon_{,x}+\frac{\partial J}{\partial q}q_{,x}+\frac{\partial J}{\partial E}E_{,x}+\frac{\partial J}{\partial \varepsilon_{,x}}\varepsilon_{,xx}+\frac{\partial J}{\partial q_{,x}}q_{,xx}+\frac{\partial J}{\partial E_{,x}}E_{,xx}\geq 0. \end{array}$$

In order to derive the consequences of such constraint on the constitutive quantities *T*, *s*, and *J*, we must substitute it in the balance Equations (69b) and (70), together with their differential consequences, (76a) and (76b). Therefore, we obtain

(79) $$\begin{array}{c} \;[\frac{\partial s}{\partial \varepsilon }\left(\lambda +2\mu \right)E-\frac{\partial s}{\partial \varepsilon }\bar{b}\varepsilon +\frac{\partial s}{\partial \varepsilon }\hat{T}+\varrho \frac{\partial s}{\partial E}+\frac{\partial s}{\partial \varepsilon_{,x}}\left(\lambda +2\mu \right)E_{,x}\\ -\frac{\partial s}{\partial \varepsilon_{,x}}\bar{b}\varepsilon_{,x}\;+\frac{\partial s}{\partial \varepsilon_{,x}}\frac{\partial \hat{T}}{\partial \varepsilon }\varepsilon_{,x}+\frac{\partial s}{\partial \varepsilon_{,x}}\frac{\partial \hat{T}}{\partial q}q_{,x}+\frac{\partial s}{\partial \varepsilon_{,x}}\frac{\partial \hat{T}}{\partial E}E_{,x}]\dot{E}\\ \;+\left[\frac{\partial s}{\partial \varepsilon_{,x}}{\frac{\partial \hat{T}}{\partial \varepsilon }}_{,x}\varepsilon_{,xx}+\frac{\partial s}{\partial \varepsilon_{,x}}\frac{\partial \hat{T}}{\partial q_{,x}}q_{,xx}+\frac{\partial s}{\partial \varepsilon_{,x}}{\frac{\partial \hat{T}}{\partial E}}_{,x}E_{,xx}\right]\dot{E}\\ \;+\left(\frac{\partial J}{\partial q_{,x}}-\frac{\partial s}{\partial \varepsilon_{,x}}\right)q_{,xx}+\left(\frac{\partial J}{\partial \varepsilon_{,x}}-\frac{\partial s}{\partial q_{,x}}\frac{\bar{\kappa }}{\tau }+\right)\varepsilon_{,xx}+\left(\frac{\partial J}{\partial E_{,x}}-\frac{\partial s}{\partial q_{,x}}\frac{\Lambda }{\tau }\right)E_{,xx}+\\ \;\left(-\frac{\partial s}{\partial \varepsilon_{,x}}\bar{b}\varepsilon +\frac{\partial s}{\partial \varepsilon_{,x}}\hat{T}+\frac{\partial s}{\partial \varepsilon_{,x}}\left(\lambda +2\mu \right)E+\varrho \frac{\partial s}{\partial E_{,x}}\right){\dot{E}}_{,x}+f\left(\Sigma \right)\geq 0, \end{array}$$

wherein f(Σ) is a suitable function defined on the state space. Thus, since in Equation (79) the higher derivatives $\left(\dot{E},\;\varepsilon_{,xx},\;E_{,xx},\;{\dot{E}}_{,x}\right)$ are independent of their coefficients and can take arbitrary values, the terms that are linear in such derivatives must vanish. Moreover, according to Theorem 7 the quadratic part in the higher derivatives must be non-negative. Then, the following theorem is true.

**Theorem** **8.** *The generalized entropy inequality (79) is satisfied whatever the thermodynamic process is if, and only if, the following thermodynamic restrictions hold:*

(80) $$\frac{\partial s}{\partial \varepsilon }\left(\lambda +2\mu \right)E-\frac{\partial s}{\partial \varepsilon }\bar{b}\varepsilon +\frac{\partial s}{\partial \varepsilon }\hat{T}+\varrho \frac{\partial s}{\partial E}+\frac{\partial s}{\partial \varepsilon_{,x}}\left(\lambda +2\mu \right)E_{,x}+$$

$$-\frac{\partial s}{\partial \varepsilon_{,x}}\bar{b}\varepsilon_{,x}+\frac{\partial s}{\partial \varepsilon_{,x}}\frac{\partial \hat{T}}{\partial \varepsilon }\varepsilon_{,x}+\frac{\partial s}{\partial \varepsilon_{,x}}\frac{\partial \hat{T}}{\partial q}q_{,x}+\frac{\partial s}{\partial \varepsilon_{,x}}\frac{\partial \hat{T}}{\partial E}E_{,x}=0,$$

(81) $$\frac{\partial J}{\partial q_{,x}}-\frac{\partial s}{\partial \varepsilon_{,x}}=0,$$

(82) $$\frac{\partial J}{\partial \varepsilon_{,x}}-\frac{\partial s}{\partial q_{,x}}\frac{\bar{\kappa }}{\tau }=0,$$

(83) $$\frac{\partial J}{\partial E_{,x}}-\frac{\partial s}{\partial q_{,x}}\frac{\Lambda }{\tau }=0,$$

(84) $$\frac{\partial s}{\partial \varepsilon_{,x}}\hat{T}+\frac{\partial s}{\partial \varepsilon_{,x}}\left(\lambda +2\mu \right)E+\varrho \frac{\partial s}{\partial E_{,x}}-\frac{\partial s}{\partial \varepsilon_{,x}}\bar{b}\varepsilon =0,$$

(85) $$\frac{\partial s}{\partial \varepsilon_{,x}}{\frac{\partial \hat{T}}{\partial \varepsilon }}_{,x}\varepsilon_{,xx}\dot{E}+\frac{\partial s}{\partial \varepsilon_{,x}}\frac{\partial \hat{T}}{\partial q_{,x}}q_{,xx}\dot{E}+\frac{\partial s}{\partial \varepsilon_{,x}}{\frac{\partial \hat{T}}{\partial E}}_{,x}E_{,xx}\dot{E}+f\left(\Sigma \right)\geq 0.$$

The last inequality can be written as follows

(86) $$\left(\begin{array}{ccc} \frac{\partial s}{\partial \varepsilon_{,x}}{\frac{\partial \hat{T}}{\partial \varepsilon }}_{,x} & 0 & 0\\ 0 & \frac{\partial s}{\partial \varepsilon_{,x}}\frac{\partial \hat{T}}{\partial q_{,x}} & 0\\ 0 & 0 & \frac{\partial s}{\partial \varepsilon_{,x}}{\frac{\partial \hat{T}}{\partial E}}_{,x} \end{array}\right)\left(\begin{array}{c} \varepsilon_{,xx}\\ q_{,xx}\\ E_{,xx} \end{array}\right)\left(\begin{array}{c} \dot{E}\\ \dot{E}\\ \dot{E} \end{array}\right)+f\left(\Sigma \right)\geq 0.$$

According to Theorem 7, the inequality above is satisfied for arbitrary values of $\varepsilon_{,xx},q_{,xx},E_{,xx}$, and $\dot{E}$ if, and only if, the matrix

$$\left(\begin{array}{ccc} \frac{\partial s}{\partial \varepsilon_{,x}}{\frac{\partial \hat{T}}{\partial \varepsilon }}_{,x} & 0 & 0\\ 0 & \frac{\partial s}{\partial \varepsilon_{,x}}\frac{\partial \hat{T}}{\partial q_{,x}} & 0\\ 0 & 0 & \frac{\partial s}{\partial \varepsilon_{,x}}{\frac{\partial \hat{T}}{\partial E}}_{,x} \end{array}\right)$$

is positive semidefinite and, moreover, f(Σ)≥0. This is possible, in principle, by assigning suitable constitutive equations for *s*, *J*, and $\hat{T}$.

The following considerations are in order:

- the restrictions above show that *T*, *J*, and, remarkably, *s* may depend on all the gradients of the unknown variables (ε,q,E);
- the dependency of the stress tensor on $E_{,x}$ has been achieved without changing the fundamental laws of thermodynamics, but it was a natural consequence of the mathematical technique applied to exploit the entropy inequality;
- the classical thermodynamic relation $\frac{\partial s}{\partial \varepsilon }=\frac{1}{\vartheta }$ leads to the conclusion that the temperature may also depend on the non-equilibrium variables $\left(q,\;\varepsilon_{,x},\;q_{,x},\;E_{,x}\right)$, as well as on the equilibrium ones, ε and *E*;
- the restrictions (81)–(83) show that the constitutive equation of the entropy flux takes a form that is more general than that postulated in [4,5,6], i.e., $J=\frac{q}{\vartheta }$.

**Remark** **9.** *The dependency of the specific entropy, s, and of the absolute temperature, $\vartheta ={\left(\frac{\partial s}{\partial \varepsilon }\right)}^{-1}$, on $\varepsilon_{,x}$ is a peculiarity of the extended procedures and cannot be achieved by applying the classical ones (see, for instance, Equation (5.2) in *[4]*, Equation (4.15) in *[5]*, and Equation (6.27b) in *[6]*). In order to see by a practical example, the subsequent changes in the mathematical structure of the theory, let us neglect for a while the dependency of all the constitutive functions and of the heat equation on $q_{,x}$ and $E_{,x}$ and suppose $\hat{T}=-\bar{b}L_{0}\varepsilon_{,x}$, where $L_{0}$ is a characteristic length. In this way, $T=\left(\lambda +2\mu \right)E-\bar{b}\varepsilon -\bar{b}L_{0}\varepsilon_{,x}$. In the linear approximation, according to which the product $\varepsilon_{,x}\dot{E}$ is negligible, the system of equations ruling the evolution of the continuous at hand now becomes*

(87a) $$\begin{array}{cc} \; & \varrho \ddot{u}-\left(\lambda +2\mu \right)u_{,xx}-\bar{b}\varepsilon_{,x}-\bar{b}L_{0}\varepsilon_{,xx}=0, \end{array}$$

(87b) $$\begin{array}{cc} \; & \varrho \dot{\varepsilon }+q_{,x}-\left[\left(\lambda +2\mu \right)E-\bar{b}\varepsilon \right]{\dot{u}}_{,x}=0, \end{array}$$

(87c) $$\begin{array}{cc} \; & \tau \dot{q}+q+\bar{\kappa }\varepsilon_{,x}=0. \end{array}$$

*It is easy to see that, due to the presence of the term $\varepsilon_{,xx}$ in Equation (87a), the system above is parabolic, although the classical Fourier law $q=-\bar{\kappa }\varepsilon_{,x}$ has been generalized with Cattaneo’s Equation (87c) *[28]*. This result sheds new light on the classical theory of linear thermoelasticity, in which it is believed that the replacement of Fourier’s law with Cattaneo’s equation is sufficient to make the system hyperbolic, which guarantees the propagation of thermomechanical waves.*

## 5. Conclusions

In continuum thermodynamics, the second law is regarded as a constraint on the constitutive equations [4]. Muschik and Ehrentraut provided a rigorous proof of such an assumption under the hypothesis that, at an arbitrary instant, $t_{0}$, in an arbitrary point, $P_{0}$, of a continuous system, the entropy production is zero if, and only if, $P_{0}$ is in thermodynamic equilibrium [11]. Recently, Cimmelli and Rogolino incorporated such an assumption into a more general formulation of the second law of thermodynamics [16].

In [17], we extended the results in [16] to the case in which there are surfaces across which the unknown fields suffer jump discontinuities. Here, we have shown that the same conclusions achieved in [11,16] hold when the classical Coleman–Noll procedure for the exploitation of the entropy inequality is generalized by also introducing into the dissipation inequality the gradient extensions of the basic equations. An extended Coleman–Noll procedure was applied to analyze the strain-gradient elasticity.

Such an investigation seems to be important for both practical and theoretical ends.

From the practical point of view, in the last decades substantial effort was dedicated to fabricating microstructured or functionally graded materials, with the scope of controlling their physical properties, [30,31]. Many of these materials are suitably described by non-local constitutive equations, whose compatibility with the second law of thermodynamics must be proven. In Section 4, we have analyzed the thermodynamic compatibility of such equations for a one-dimensional thermoelastic solid with the gradient of the strain entering the state space. Such an analysis has been carried out without modifying the classical forms of the first and second laws of thermodynamics.

From a theoretical point of view, the present investigation leads to a deeper view of the role of the second law of thermodynamics in the presence of generalized exploitation procedures, in which some differential consequences of the basic balance laws are also considered as constraints to be substituted into the entropy inequality. In general, for arbitrary weakly non-local constitutive equations, it is important to investigate if the thermodynamic compatibility makes it necessary to modify the basic thermodynamic laws or not.

In future research, we aim at extending the present results by considering different nanostructured systems such as, for instance, non-local thermoelectric heat conductors or thermal rectifiers.

## Data Availability

No new data were created or analyzed in this study. Data sharing is not applicable to this article.
